# Conference abstracts – Liberating Methadone: Building a Roadmap & Community for Change

**DOI:** 10.1186/s13722-025-00535-4

**Published:** 2025-02-27

## P1 A novel hand-held personalized dual biometrically controlled device for at-home dispensation of methadone

### John Timberlake, MBA^1^, Thomas B. King^1^, Christy Corey^1^, John Kirkpatrick^1^

#### ^1^Berkshire Biomedical, LLC, Austin, Texas, United States

##### **Correspondence:** John Timberlake (jtimberlake@berkbiomed.com)

*Addiction Science & Clinical Practice* 2025, **20(Suppl 1)**:P1

Over 2.7 million people in U.S. have an opioid use disorder (OUD), but only 550,000 people receive liquid methadone to treat OUD. Methadone treatment, by law, is only accessible from federally certified opioid treatment programs (OTPs). Methadone has inherent limitations to treatment, one of which includes the need of most patients to receive supervised methadone dosing at a clinic location. This problem is compounded as patients may be enrolled in clinics far from their home, where transportation becomes a time and cost burden, making it challenging to maintain employment or meet family responsibilities. At-home dosing systems may reduce these burdens on patients and clinics. To be safe for patients, take-home therapy should minimize the opportunity for misuse and diversion of methadone and would ideally provide confirmation to the healthcare provider that the intended patient has received each of their doses. Missing or overuse of methadone doses may result in relapse, opioid withdrawal syndrome, or overdose. An easy-to-use at-home automated dosing and remote monitoring system with enhanced security features may resolve these challenges, maintaining increased flexibility for patients receiving methadone. The Computerized Oral Prescription Administration System (COPA™) is a hand-held, personalized, automated dispensing system currently under development, which has not been reviewed by the US FDA. It is designed to deliver oral liquid medications to an Authenticated Intended User (AIUTM) upon confirmation of dual biometrics at each dose via fingerprint and dentition. The system allows for dosages of medication to be dispensed only at prescribed times and volumes, along with real-time monitoring with data access for tracking and analysis. Conclusion: COPA™ is uniquely suited for at-home delivery of methadone to only the AIU. COPA may reduce barriers for OUD patients seeking methadone, expand the number of patients who could utilize at-home methadone and reduce the potential for inappropriate use of methadone.

**Acknowledgements**

This program is supported by the National Institute on Drug Abuse of the National Institutes of Health under Award Number R44DA057185. Melissa Olivadoti contributed to the medical writing, editing, and creation of the abstract and poster.

## P2 Changes in Canadian methadone practices during COVID-19: a community led survey

### Adam Palayew^1^, Matthew Bonn^2^, David Frank^3^, Louise Vincent^4^, Frank Chrichlow^2^, Natasha Touesnard^2^, Sarah Brothers^5^

#### ^1^Department of Epidemiology, University of Washington, Seattle, Washington, United States; ^2^Canadian Association of People Who Use Drugs, Darmouth, Nova Scotia, Canada; ^3^New York University, New York, New York, United States; ^4^North Carolina Survivors Union, Greensboro, North Carolina, United States; ^5^Pennsylvnia State University, State College, Pennsylvania, United States

##### **Correspondence:** Adam Palayew (apalayew@gmail.com)

*Addiction Science & Clinical Practice* 2025, **20(Suppl 1)**:P2

Objective: We described changes in methadone during early COVID-19 with data from Canadian methadone patients. We aimed to assess if fewer methadone treatment restrictions were associated with increased economic stability and autonomy. Methods: We conducted an online survey from 09-02-2020 to 02–04-2021 with members of the Canadian Association of People who use Drugs (CAPUD). People self-selected into the survey. The survey was developed by members of CAPUD and the National Survivors Union. Questions pertained to demographics and methadone treatment experiences before and during COVID-19. Results: In total 97 individuals responded. The most common age groups were 25–30 (n = 22), 31–35 and 51 + (n = 18 each), and 41–45 (n = 13). Most of the sample (n = 55) were women. Most people identified as white (n = 81), with 14 people identifying as non-white of whom nine were Indigenous. Most of the sample lived in urban areas (n = 72) and 20 were from rural areas. For changes in methadone practices, pre-COVID-19, 31 people reported daily witnessed dosing, whereas 19 people reported daily witnessed dosing during COVID-19. Before COVID-19, 20 people reported no mandatory drug screening with 48 people reporting no mandatory drug screening during COVID-19. During the pre-COVID-19-period, 42 people reported not getting any carries, this number decreased to 30 people during COVID-19. 30 people received more carries, 63 reported no change, and 4 people received fewer. Among those who got more carries 14 reported an improvement in their economic stability and 25 reported more control over their time. Among the four people who got less carries one said it worsened the control over their time significantly and the three other people discontinued methadone. Conclusions: People in methadone treatment during COVID-19 reported a range of experiences on how methadone dispensing changed. Those who received an increase in carries reported increased economic stability as well as more control over their time.

## P3 SI-CBPAR: toward structural indicators of community based participatory action research

### Beth E. Meyerson^1^, Irene Garnett^1^, Danielle Russell^2^, Arlene Mahoney^3^, Savannah Samorano^1^

#### ^1^Drug Policy Research and Advocacy Board, Harm Reduction Research Lab, Family and Community Medicine, University of Arizona College of Medicine-Tucson, Tucson, Arizona, United States; ^2^Hepatitis and Drug Use Research Group, Kirby Institute, University of New South Wales, Sydney, New South Wales, Australia; ^3^Southwest Recovery Alliance, Phoenix, Arizona, United States

##### **Correspondence: **Beth E. Meyerson (bmeyerson@arizona.edu)

*Addiction Science & Clinical Practice* 2025, **20(Suppl 1)**:P3

Meyerson BE, Russell DM, Mahoney A, Garnett I, Samorano S. SI-CBPAR: Towards structural indicators of community-based participatory action research. *Drug Alcohol Rev*. 2024; 43(5): 1049–1061. 10.1111/dar.13764

## P4 Drug policy research and advocacy board (DPRAB): sending MAT to rehab-transforming MOUD through community-focused research & evidence-based advocacy

### Irene Garnett^1^, Beth Meyerson^1^, Members of Drug Policy Research and Advocacy Board^1^

#### ^1^Drug Policy Research and Advocacy Board (DPRAB), Harm Reduction Research Lab, University of Arizona College of Medicine-Tucson, Phoenix, Arizona, United States

##### **Correspondence:** Irene Garnett (ireneygarnett@hotmail.com)

*Addiction Science & Clinical Practice* 2025, **20(Suppl 1)**:P4

This is a conversation about the DPRAB so that others can 'try this at home' or discuss what user-centered treatment looks like, what is working currently, and what needs rehabbing. What it is: Arizona-based statewide transdisciplinary group of (MOUD) providers, MOUD users, people with living/lived drug use experience, harm reduction organizations, state Medicaid professionals, and university researchers. Collaboration between providers and people who use drugs and/or MAT seeking to rehabilitate OTPs into flexible, patient-centered models that empower users and reduce stigma. What we do: DPRAB drives the research out of the University of Arizona Harm Reduction Research Lab, which provides resources and funding. The DPRAB has mobilized its members and hired from the community to conduct several studies intended to improve MAT by demonstrating change is needed (gathering interviews regarding methadone access during COVID, a secret shopper study of MOUD providers, etc.) and making change (influencing state policy to assure access to multiday dosing by advocating with the SOTA and with providers and patients). The DPRAB published several papers together and drafted and circulated a memo to increase awareness about multiday methadone dosing regulations in response to a letter from the SOTA. These recommendations are what drive the university to seek grants to fund additional studies promoting improved MAT (MPACT, OPTIC). What is good about it: We will discuss the micro, mezzo, and macro benefits for members of the DPRAB as well as policy 'wins.'. What should change? Over time we have improved training for all members to feel empowered to engage. We continue to interrogate the power dynamics between provider and patient members of the DPRAB.

**Acknowledgements**

We acknowledge the member of the Drug Policy Research and Advocacy Board (DPRAB) and people who use drugs in Arizona.

## P5 Experiences of intersectional stigma and aging with opioid use disorder: a qualitative analysis

### Katie Fitzgerald Jones^1^, Jonathan F. Bean^1^, Mirella A. Orozco^2^, Mari Miyoshi^3^, Heidi Doland^2^, Alison A. Moore^4^

#### ^1^New England Geriatric Research Education and Clinical Center (GRECC), VA Boston Healthcare System, Boston, Massachussetts, United States; ^2^Division of Geriatrics, Gerontology, and Palliative Care, University of California San Diego, San Diego, California, United States; ^3^Department of Psychiatry, University of California San Diego, San Diego, California, United States; ^4^Deparment of Medicine, University of California San Diego, San Diego, California, United States.

##### **Correspondence: **Katie Fitzgerald Jones (katie.jones4@va.gov)

*Addiction Science & Clinical Practice* 2025, **20(Suppl 1)**:P5

Aim: The number of older adults entering opioid treatment programs (OTPs) for treatment of opioid use disorder (OUD) is increasing sharply in the US. This population has a high prevalence of chronic disease and acute healthcare utilization. We explored the experience of aging with OUD and barriers to medical care for older adults who receive care in OTPs. Methods: From November 2021 to July 2022, we conducted 1-to-1, semi-structured qualitative interviews with 36 adults aged 55 and older enrolled in OTPs in San Diego, California. Interviews were conducted in English or Spanish, audio-recorded, transcribed, systematically coded, and analyzed to identify key themes regarding the challenges of aging with OUD and managing chronic diseases. Results: Participants had a mean age of 63.4 (SD 5.1) years, 11 (30.6%) were women, 18 (50%) identified as Hispanic/Latino, 14 (39%) as Black, and the mean current duration of methadone treatment of 5.6 years. Chronic diseases were common, with 21 (58.3%) reporting hypertension, 24 (66.7%) chronic pain, 9 (25%) untreated hepatitis C, and 32 (88.9%) having 2 or more chronic diseases. Three major themes emerged: (1) avoidance of medical care due to multiple intersectional stigmas including those related to drug use, substance use disorder treatment, ageism, and homelessness; (2) increasing isolation with aging and loss of family and peer groups; (3) urgent need for integrating medical and aging-focused care with OUD treatment in the setting of increasing health and functional challenges. Conclusions: Older adults with OUD reported increasing social isolation and declining health while experiencing multilevel stigma and discrimination. The US must transform how OTPs operate to deliver care that integrates evidence-based geriatric models of medical care incorporated with OUD treatment. Such integrated care must address the lifelong and intersectional stigma.

**Acknowledgements**

This study would not be possible without the support of Luke Bergman PhD and Melanie Briones, MPH, MBA, and approval of Behavioral Health Services of the County of San Diego. We thank the study participants for their time and willingness to share their experiences. Finally, we thank Michele G Shedlin PhD for her mentorship and feedback for the design of this study and the interview guide. Funding This study was funded by grants from the National Institute on Health: K23DA043651 and T35AG026757-19.

## P6 Emerging trends in xylazine use: an integrative review of prevalence, risks, and geographic distribution

### Evans Kyei^1^, Lingling Zhang^1^, Suzanne Leveille^1^

#### ^1^Department of Nursing, University of Massachusetts Boston, Boston, Massachusetts, United States

##### **Correspondence:** Evans Kyei (evanskyei80@gmail.com)

*Addiction Science & Clinical Practice* 2025, **20(Suppl 1)**:P6

Background: Xylazine, an increasingly prevalent animal tranquilizer infiltrating the unregulated drug supply, poses substantial public health concerns. This integrative review synthesizes existing evidence on the prevalence, risks, and geographic distribution of xylazine within the illicit drug market. Methods: A comprehensive search across PubMed, CINAHL, and PsycINFO databases yielded 321 relevant studies. After rigorous screening, we included 13 studies, incorporating various research designs. The GRADE system categorized ten studies as high quality and three as moderate quality. Results: Our findings underscore the escalating presence of xylazine in tandem with other substances, heightening the risk of fatal outcomes. Addressing this crisis necessitates tailored interventions for high-risk populations. Unique characteristics of xylazine, including its potentiation of opioid effects and limited responsiveness to naloxone, demand innovative emergency response strategies and alternative treatments. Geographic distribution patterns reveal its widespread prevalence, underscoring the urgency of robust monitoring efforts and routine testing. Conclusion: This review emphasizes the imperative for evidence-based strategies to combat the rising tide of xylazine-related harm. Interdisciplinary collaboration is pivotal in developing targeted interventions that can mitigate its prevalence and adverse effects. Through dedicated research, vigilant surveillance, comprehensive education, and proactive harm reduction initiatives, public health can be shielded from the detrimental impact of xylazine. Policy Implications: In terms of policy implications, urgent action is imperative. Policies must be enacted to regulate the availability of xylazine, bolster surveillance mechanisms, and facilitate the development of precise interventions aimed at mitigating the risks associated with its illicit use. These proactive measures are essential in safeguarding public health and curbing the adverse effects of xylazine in the illicit drug market. Keywords: xylazine, illicit drug market, policy implications, harm reduction.

**Acknowledgements**

Rappaport Institute of Greater Boston, Harvard Kennedy School, Cambridge, MA Office of Recovery Services, Boston Public Health Commission, Boston, MA.

## P7 Assessing gaps in continuation of methadone maintenance therapy for hospitalized patients with opioid use disorder

### Meaghan Coyne, MD^1^, Katherine Mullins MD^2^

#### ^1^Population Health, NYU Langone Health, Brooklyn, New York, United States; ^2^Department of Medicine, Family Health Centers at NYU Langone Health, Brooklyn, New York, United States

##### **Correspondence: **Meaghan Coyne (meaghan.coyne@nyulangone.org)

*Addiction Science & Clinical Practice* 2025, **20(Suppl 1)**:P7

The practice of confirming outpatient methadone doses for hospitalized patients enrolled in Opioid Treatment Programs (OTPs) is a widely utilized safety measure to prevent in-hospital overdose. The NYU Langone Hospital inpatient pharmacy prevents administration of methadone doses greater than 20 mg until the treatment team verifies a patient’s home dose with their OTP. The extent to which this practice contributes to treatment delays within our hospital system is not known. We conducted a retrospective chart review of inpatients on methadone to assess the time from admission to first dose and to resumption of home dose. We included inpatients admitted to the NYU Langone system between October and December of 2022 who received methadone. We excluded patients who were started on methadone in the hospital, either for treatment of opioid use disorder or for opioid withdrawal. Of 58 patients who met inclusion criteria, 79% (n = 46) received their home dose, 3% (n = 2) received a higher dose, and 17% (n = 10) received lower doses, with an average decrease in dose of 44 mg. We measured the time from admission to receipt of > 20 mg of methadone as a proxy for the time to home dose verification. On average, the time from admission to a dose > 20 mg was 30.4 h, with a range of 1.2 h to 96 h. Four patients never received doses > 20 mg. These results suggest that gaps persist in the continuation of home methadone doses in the hospital setting for some patients. Limitations of our study include small sample size and lack of assessment of reasons for dose delays or reductions. Delays in OUD treatment for hospitalized patients contribute to adverse outcomes including increased rates of withdrawal and discharge against medical advice (Lail 2018), so factors that contribute to these gaps merit further investigation."

## P8 Why diverted methadone is a safe choice for me

### Keri Ballweber^1*^

#### ^1^Oak Park, Illinois, United States

##### **Correspondence:** Keri Ballweber (keriharmredux@gmail.com)

*Addiction Science & Clinical Practice* 2025, **20(Suppl 1)**:P8

Patients on methadone programs are inherently vulnerable to the whims of others who have autonomy over our lives. As someone that presents as female who worked in the sex industry it is worse. I was the recipient of continual sexual harassment from staff. I was told my (legal) job wasn’t “work” so my paychecks couldn’t be used as income verification and thus I was unable to qualify for reduced fees. My counselor who was also the program’s clinical director repeatedly maligned my choice of profession and often told me my husband did not love me if he allowed me to engage in sex work. When I was sexually harassed by other patients in front of staff while wearing shorts in the height of the summer heat, I was told I was a distraction to the male patients and I must change how I dress or I would not be medicated; nothing was said to the patients who had harassed me. As someone who is a survivor of sexual abuse and who has experienced gender-based violence both in and out of an OTP setting, the cameras present in the bathrooms were a triggering ordeal, my request for a monitored oral swab drug test were denied due to the increased cost of such tests. Though I was told that the clinic would honor my request for a female or femme presenting counselor, the reality was that I often had to meet with male counselors as part of my program compliance. These plus many other reasons are why I have found breaking the law and taking diverted methadone to be the safest form of treatment for myself and others.

## P9 Changes in county-level access to medications for opioid use disorder after Medicare coverage of methadone treatment began

### Samantha J Harris^1^, Courtney R Yarbrough^2^, Amanda J Abraham^3^

#### ^1^Johns Hopkins University, Baltimore, Maryland, United States; ^2^Emory University, Atlanta, Georgia, United States; ^3^University of Georgia, Athens, Georgia, United States

##### **Correspondence: **Samantha J Harris (samanthaharris@jhu.edu)

*Addiction Science & Clinical Practice* 2025, **20(Suppl 1)**:P9

Harris, S. J., Yarbrough, C. R., & Abraham, A. J. (2023). Changes In County-Level Access To Medications For Opioid Use Disorder After Medicare Coverage Of Methadone Treatment Began: Study examines changes in county-level access to opioid use disorder medications after Medicare coverage expanded to include methadone treatment. *Health Affairs*, *42*(7), 991–996.

## P10 Use of non-prescribed medication for opioid use disorder and associated health behaviors: a preliminary analysis in adults from Baltimore who have injected drugs

### Kenneth A. Feder^1^, Becky L. Genbergy^2^

#### ^1^Department of Mental Health, Johns Hopkins Bloomberg School of Public Health, Baltimore, Maryland, United States; ^2^Department of Epidemiology, John’s Hopkins Bloomberg School of Public Health, Baltimore, Maryland, United States

##### **Correspondence: **Kenneth A. Feder (kfeder1@jh.edu)

*Addiction Science & Clinical Practice* 2025, **20(Suppl 1)**:P10

Background. Concerns about diversion of methadone and buprenorphine have historically motivated unique restrictions on the prescription and dispensing of these medications (1). However, qualitative studies show people who use non-prescribed buprenorphine and methadone say they usually do so to prevent opioid withdrawal while avoiding more dangerous drugs (2–7). Few studies have rigorously examined the behavioral and physical health effects of using non-prescribed methadone and buprenorphine. This preliminary analysis examines correlates of and health outcomes associated with non-prescribed methadone and buprenorphine use in a cohort of adults from Baltimore City who have injected drugs. Methods. Participants (n = 231) are enrolled in the AIDS Linked to the Intravenous Experience (ALIVE) study: an active community-recruited cohort of adults who have injected drugs and live in or near Baltimore (8). Participants attend twice-annual study visits to complete interviews assessing past-six-month drug use behaviors and related social determinants of health. Since March 2023, this interview has included questions about use of non-prescribed methadone or buprenorphine. We tested behavioral and demographic covariates for association with non-prescribed use of either of these medications using Fisher’s exact test. We examined the association of using non-prescribed methadone and buprenorphine with self-reported a) injecting drugs, b) non-fatal overdose, and c) sex without a condom, using unadjusted and adjusted logistic regression. Results. Fentanyl use (p = 0.049) was positively associated and cocaine use (p = 0.014) negatively associated with non-prescribed use of methadone or buprenorphine. Non-prescribed buprenorphine or methadone use was associated with reduced odds of sex without a condom (adjusted odds ratio 0.11, 95% confidence interval 0.02—0.56) Implications. Findings from this small sample are too preliminary to interpret. Data collection is ongoing. With a larger sample, we hope empirical evidence about health outcomes associated with non-prescribed use of methadone and buprenorphine can inform policies governing dispensing and prescribing those medications.

**Acknowledgements**

Grant support for ALIVE was provided by the National Institute on Drug Abuse (U01-DA-036297) This publication resulted (in part) from research supported by the Johns Hopkins University Center for AIDS Research, an NIH funded program (1P30AI094189), which is supported by the following NIH Co-Funding and Participating Institutes and Centers: NIAID, NCI, NICHD, NHLBI, NIDA, NIA, NIGMS, NIDDK, NIMHD.

**References**

1. U.S. Drug Enforcement Administration, Diversion Control Division. National forensic laboratory information system: NFLIS-drug 2020 annual report [Internet]. Springfield, VA; 2021 [cited 2022 Aug 4]. Available from: https://www.nflis.deadiversion.usdoj.gov/publicationsRedesign.xhtml
2. Genberg BL, Gillespie M, Schuster CR, Johanson CE, Astemborski J, Kirk GD, et al. Prevalence and correlates of street-obtained buprenorphine use among current and former injectors in Baltimore, Maryland. Addict Behav. 2013 Dec 1;38(12):2868–73.
3. Monte AA, Mandell T, Wilford BB, Tennyson J, Boyer EW. Diversion of Buprenorphine/Naloxone Coformulated Tablets in a Region with High Prescribing Prevalence. J Addict Dis. 2009 Jul 31;28(3):226–31.
4. Bazazi AR, Yokell M, Fu JJ, Rich JD, Zaller ND. Illicit Use of Buprenorphine/Naloxone Among Injecting and Noninjecting Opioid Users. J Addict Med. 2011 Sep;5(3):175–80.
5. Johanson CE, Arfken CL, di Menza S, Schuster CR. Diversion and abuse of buprenorphine: Findings from national surveys of treatment patients and physicians. Drug Alcohol Depend. 2012 Jan 1;120(1):190–5.
6. Johnson B, Richert T. Non-prescribed use of methadone and buprenorphine prior to opioid substitution treatment: lifetime prevalence, motives, and drug sources among people with opioid dependence in five Swedish cities. Harm Reduct J. 2019 May 2;16(1):31.
7. Feder KA, Harris SJ, Byrne L, Miller SM, Sodder S, Berman V, et al. Attitudes and beliefs about Vermont’s 2021 buprenorphine decriminalization law among residents who use illicit opioids. Drug Alcohol Depend. 2023 Jul 6;110879.
8. Vlahov D, Anthony JC, Munoz A, Margolick J, Nelson KE, Celentano DD, et al. The ALIVE study, a longitudinal study of HIV-1 infection in intravenous drug users: description of methods and characteristics of participants. NIDA Res Monogr. 1991;(109):75–10

## P11 Creating evidence based best practice resources for substance use counselors

### Cailyn Green^1^

#### ^1^School of Human Services, SUNY Empire State University, Saratoga, New York, United States

##### **Correspondence:** Cailyn Green (cailyn.green@sunyempire.edu)

*Addiction Science & Clinical Practice* 2025, **20(Suppl 1)**:P11

The purpose of this research was to identify what types of resources would support addiction counselors in performing their job duties. Counselors often must jump in and facilitate a group counseling session with little to no time for prep. This causes stress and creates pressure to come up with a clinical group activity in little time. Counselors have many job duties, and these wide arrays of responsibilities make time a limited commodity for addiction counselors. These 15 qualitative interviews focused on identifying what types of resources would support addiction counselors in doing their jobs easier and more effectively. Common themes that emerged included a set book of resources to guide counseling groups would be helpful and it would help move the groups along in a positive manner. The interviews are guiding the researcher towards creating an open education resource (OER) of group activities for addiction counselors to utilize.

## P12 Unlocking methadone: increased take-home doses of methadone improves quality of life and effectiveness of treatment from the patient perspective

### Sara Lorenz Taki, MD^1^, Ashley Magnussen^1^, Caitlyn Romano^1^, Gretchen Grappone^1^

#### ^1^Greenwich House Center for Healing, Greenwich House, New York, New York, United States

##### **Correspondence: **Sara Lorenz Taki (staki@greenwichhouse.org)

*Addiction Science & Clinical Practice* 2025, **20(Suppl 1)**:P12

Background and Aims: U.S. Regulatory changes allowed for additional methadone take-homes following Covid-19 onset. However, little is known about how dispensing trajectories changed and which factors drove variation in dispensing regimens. We 1) examined daily methadone dispensing trajectories of patients initiating treatment across 9 U.S. sites before and following regulatory changes, and 2) explored individual and site-level factors associated with dispensing trajectories. Design: Data were manually extracted from opioid treatment program (OTP) electronic health records (EHR) of methadone patients newly admitted to treatment in the year before (N = 328) and after reforms (N = 376). We used State Sequence Analysis and multifactor discrepancy analysis to identify covariates that predicted different dispensing trajectories. We visualized dispensing trajectories using regression trees. Setting and participants: Adult methadone patients newly admitted to 9 OTPs across 9 U.S. states who were followed for 6 months. Measurements: Type of daily methadone medication encounter; OTP site; cohort (pre and post Covid-19); substance use; sociodemographics. Results: Following COVID-19 regulatory changes, allotted methadone take-home doses increased from 3.5% to 13.8% of total patient-days of treatment engagement within the first six months of care. Treatment site was the observed covariate explaining more differences between trajectories, accounting for 6.2% and 9.5% of the discrepancy between sequences pre and post Covid-19. Methamphetamine users had a sharper increase in take-homes than non-users (from 3.7% to 21.2% versus 3.5% to 12.5% respectively) and higher discontinuation regardless of the cohort. After Covid-19 participants experiencing houselessness presented a higher proportion of missed doses and.

less time engaged in treatment. Conclusion: Daily methadone dispensing trajectories may depend more on treatment site practices than individual patient characteristics or federal policies. Further research into variable take-home allowances across clinics should be explored as potential targets for reducing treatment burden and improving equitable practices across methadone treatment programs.

## P13 What influences methadone dispensing? analyzing daily trajectories of methadone dispensing among patients admitted to U.S. opioid treatment programs (OTPs) before and after COVID-19 using state sequence analysis

### Ignacio Borquez^1^, Arthur Robin Williams^2^, Mei-Chen Hu^3^, Lexa Harpel^3^, Nicole Aydinoglo^3^, Magdalena Cerdá^1^, John Rotrosen^1^, Edward V. Nunes^3^, Noa Krawczyk^1^

#### ^1^Center for Opioid Epidemiology and Policy, Department of Population Health, NYU Grossman School of Medicine, New York, New York, United States; ^2^Department of Psychiatry, Columbia University, New York, New York, United States; ^3^New York State Psychiatric Institute, New York, New York, United States

##### **Correspondence:** Ignacio Borquez (ignacio.borquez@nyulangone.org)

*Addiction Science & Clinical Practice* 2025, **20(Suppl 1)**:P13

Background and Aims: U.S. Regulatory changes allowed for additional methadone take-homes following Covid-19 onset. However, little is known about how dispensing practices changed and which factors drove variation in take-home regimens. We 1) examined methadone take-home trajectories of patients initiating treatment across 9 U.S. sites before and following regulatory changes, and 2) explored individual and site-level factors associated with dispensing trajectories. Design: Data were manually extracted from opioid treatment program (OTP) electronic health records (EHR) of methadone patients newly admitted to treatment in the year before (N = 317) and after reforms (N = 363). We used State Sequence Analysis and multifactor discrepancy analysis to identify covariates that predicted different dispensing trajectories. We visualized dispensing trajectories using regression trees. Setting and participants: Adult.

methadone patients newly admitted to 9 OTPs across 9 U.S. states who were followed for 6 months. Measurements: Frequency and type of daily methadone medication encounter; OTP site; cohort (pre and post Covid-19); substance use and sociodemographics. Results: Following COVID-19 regulatory changes, allotted methadone take-home doses increased from 3.1% to 13.8% of total patient-days of treatment engagement within the first six months of care. Treatment site was the most relevant covariate explaining differences between trajectories, which accounted for 5.8% of the variation, followed by cohort (pre vs. post Covid-19) (1.3%) and type of insurance (0.9%). Cohort was the main factor explaining changes in dispensing across six sites. Methamphetamine users had a sharper increase in take-homes than non-users (from 3.7% to 20.5% versus 3% to 12.1% respectively). Methamphetamine use and unemployment increased discontinuation regardless of the cohort. Conclusion: Methadone take-home dosing patterns may depend more on treatment site practices than individual patient characteristics or federal policies. Further research into variable take-home allowances across clinics should be explored as potential targets for reducing treatment burden and improving equitable practices across methadone treatment programs.

**Acknowledgements**

NIDA Clinical Trials Network 0112 UG1 DA013035-20; Dr. Krawczyk was supported by the National Institute on Drug Abuse of the National Institutes of Health under Award Number K01DA055758. The content is solely the responsibility of the authors and does not necessarily represent the official views of the National Institutes of Health.

## P14 Adoption of methadone take home policy by U.S. state opioid treatment authorities during COVID-19

### Victor Roy, MD, PhD^1^, Michele Buonora, MD, MS, MHS^1^, Caty Simon^2^, Bridget Dooling, JD^3^, Paul Joudrey, MD, MPH^4^

#### ^1^Yale National Clinician Scholars Program, Yale School of Medicine, New Haven, Connecticut, United States; ^2^Whose Corner Is it Anyway, National Survivors Union, Holyoke, Massachusetts, United States; ^3^Moritz College of Law, Ohio State University, Columbus, Ohio, United States; ^4^Division of General Internal Medicine, Center for Research on Health Care, University of Pittsburgh School of Medicine, Pittsburgh, Pennsylvania, United States

##### **Correspondence:** Victor Roy (victor.roy@yale.edu)

*Addiction Science & Clinical Practice* 2025, **20(Suppl 1)**:P14

Roy V, Buonora M, Simon C, Dooling B, Joudrey P. Adoption of methadone take home policy by U.S. state opioid treatment authorities during COVID-19. *International Journal of Drug Policy*. 2024;124:104302. 10.1016/j.drugpo.2023.104302

## P15 Methadone prescribing by addiction specialists likely to leave communities without available methadone treatment

### Paul Joudrey^1^, Dylan Halpern^2^, Qinyun Lin^3^, Susan Paykin^2^, Christina Mair^4^, Marynia Kolak^5^

#### ^1^Internal Medicine, University of Pittsburgh, Pittsburgh, Pennsylvania, United States; ^2^Data Science Institute, University of Chicago, Chicago, Illinois, United States; ^3^Institute of Medicine, University of Gothenburg, Gothenburg, Sweden; ^4^Departmen of Behavioral and Community Health Sciences, University of Pittsburgh School of Public Health, Pittsburgh, Pennsylvania, United States; ^5^Department of Geography and GIScience, University of Illinois, Urbana, Illinois, United States

##### **Correspondence: **Paul Joudrey (pjoudrey@pitt.edu)

*Addiction Science & Clinical Practice* 2025, **20(Suppl 1)**:P15

Paul J Joudrey, Dylan Halpern, Qinyun Lin, Susan Paykin, Christina Mair, Marynia Kolak, Methadone prescribing by addiction specialists likely to leave communities without available methadone treatment, *Health Affairs Scholar*, Volume 1, Issue 5, November 2023, qxad061. 10.1093/haschl/qxad061

## P16 Take-home expansion scope and impact study (THESIS): methodology for health services research investigation of practice changes in methadone treatment

### Jan Gryczynski^1^, Tami Mark^2^, Gary Zarkin^2^, Nicholas Van Dyke^3^, Karli Hochstatter^1^, Courtney D. Nordeck^1^, Mishka Terplan^1^, Robert P. Schwartz^1^

#### ^1^Social Research Center, Friends Research Institute, Baltimore, Maryland, United States; ^2^RTI International, Durham, North Carolina, United States; ^3^BarMark Health Services, Lewisville, Texas, United States

##### **Correspondence: **Jan Gryczynski (jgrycznski@friendsresearch.org)

*Addiction Science & Clinical Practice* 2025, **20(Suppl 1)**:P16

Since the initial approval of methadone maintenance treatment in the US over 50 years ago, federal regulations have required frequent clinic attendance to administer methadone to reduce the risks of methadone diversion. Patients could only receive take-home methadone after significant time in treatment while meeting rigid standards for adherence and stability. However, these regulations were not grounded in strong empirical evidence. In response to the COVID-19 pandemic, SAMHSA swiftly permitted states to apply for exemptions that expanded availability of take-home methadone. Opioid Treatment Programs (OTPs) were suddenly able to dispense up to 14 days of take-home methadone for ‘less stable’ patients, and 28 days for ‘stable’ patients. Subsequently, SAMHSA reaffirmed the regulatory exemptions, building momentum for permanent regulatory reform. Research is needed to examine the scope of these major changes to care delivery, the extent to which they are equitably implemented to promote treatment access and patient-centered care, and their impact on clinical outcomes. This presentation will describe an emerging study of methadone treatment services conducted with support from the National Institute on Drug Abuse by Friends Research Institute, RTI International, and BayMark Health Services. The study will draw upon multiple data sources, including OTP clinical records from BayMark, the largest provider of outpatient OUD treatment.

in the U.S., spanning 115 OTPs across 27 states and the District of Columbia. This study will leverage data from BayMark to track OTP practices over time, examine the relationship of expanded take-home methadone with patient outcomes, and develop predictive models to inform clinical decision-making. All analyses will consider health equity and examine disparities with respect to patients’ sex, race, and ethnicity. The study is poised to provide novel data on how OTPs implement their new discretionary powers to expand access to take-home methadone, and which patients benefit most from these new flexibilities.

**Acknowledgements**

NIDA R01 DA057052

## P17 Liberating methadone for people returning to the community from Rikers island

### Michael Matteo^1^, Raji Edayathumangalam^2^

#### ^1^Opioid Treatment Program, New York City Health and Hospitals Corporation, Correctional Health Services, New York, New York, United States; ^2^Department of Policy, New York County Defenders Services, New York, New York, United States.

##### **Correspondence: **Michael Matteo (matteom@nychhc.org)

*Addiction Science & Clinical Practice* 2025, **20(Suppl 1)**:P17.

Each day, approximately 600 persons out of just under 6000 incarcerated within the NYC jail system at Rikers Island receive methadone to help treat moderate to severe opioid use disorder. KEEP, the substance use treatment division and SAMSHA-certified opioid treatment program within NYC Health and Hospitals Corporation’s Correctional Health Services, has been providing methadone to patients on Rikers Island since the 1980s. KEEP offers all three FDA-approved medications for opioid use disorder (MOUDs): methadone, buprenorphine, and naltrexone. However, methadone remains the medication of choice for most, with approximately 75% of KEEP-enrolled patients opting for this treatment. All patients receiving methadone are provided with an aftercare referral to a community-based OTP for continued treatment upon jail release. Nevertheless, significant barriers to continuation of care exist. These include a lack of community OTPs that provide evening and weekend medication for unexpected weekend discharges, delays due to a fragmented intake process at OTPs even for patients who have an aftercare referral, challenges associated with travel restrictions for those under community supervision (electronic monitoring), limited resources for uninsured individuals seeking methadone treatment, and obstacles arising from inadequate housing access, transportation, and other basic psychosocial needs. To address these barriers, the implementation of supplementary community resources and support for MOUD patients during the transitional period following release from Rikers is crucial. This may involve expanding OTP service hours and locations; revising regulations to permit community pharmacies to dispense one or two bridging methadone doses; and fostering collaboration between courts, defense services, and jail-based clinical staff to facilitate seamless jail releases and care continuation in the community for individuals receiving MOUD. A review and revision of current federal opioid treatment standards, which include stigmatizing language and pose challenges to providing “take-home” methadone doses for individuals returning to the community from incarceration settings, may also be beneficial.

## P18 “I felt I took nothing”: patients’ perspectives on methadone initiation in the fentanyl era

### Eva DeLappe^1^, Julia Shi, MD^2^, Kimberly Sue, MD, PhD^2^

#### ^1^CHAMP Training Program, Yale Primary Care Residency Program, New Haven, Connecticut, United States; ^2^Addiction Medicine, APT Foundation and Yale School of Medicine, New Haven, Connecticut, United States

##### **Correspondence: **Eva DeLappe (eva.delappe@yale.edu)

*Addiction Science & Clinical Practice* 2025, **20(Suppl 1)**:P18

Objectives: We aimed to assess patients' experiences around methadone initiation dosing in the fentanyl era. Background: Clinicians suspect methadone initiation needs to adjust to fentanyl. Many initiation protocols draw on data from the 1960s, when the less-potent heroin predominated. (1) Observers have noted that patients starting methadone continue to use fentanyl to prevent withdrawal. (2) Many require higher doses to achieve therapeutic response. (3) Clinicians have offered alternative initiation schedules to meet patients' high opioid tolerance and keep patients safe from overdose or toxicity. (4) Little is known about patients' perspectives on the matter. Methods: We gathered a sample of patients' perspectives through anonymous, retrospective phone surveys. Our population was 72 patients with OUD who completed methadone intakes at an urban OTP (3650 patient census) from November to December 2022. 10 participants completed the phone survey. Data was evaluated through thematic analysis. Results: 90% of participants found methadone helpful. All used illicit opioids daily (20% oral, 80% IV), with IV use in bundles ranging from <1 (30%), 1–2 (20%), and 2 (30%). On Day 1, 60% experienced withdrawal after taking 30 mg. 50% used fentanyl to feel better, and 33% wanted to but were in a facility. 70% thought they would need to start at 50 mg to prevent withdrawal, with doses ranging from 50 mg-100 mg. 50% believed starting at a higher dose would make them feel stable and reduce illicit use faster. As of 7/14/23, OTP retention data showed 50% are inactive; 40 mg was the median last dose and 61% were discharged within 3 months. Conclusions: Most patients think a higher starting dose of methadone is needed in the fentanyl era. Limitations include a small response size; many of the non-responders might have returned to fentanyl use. Future projects could consider implementing novel initiation protocol and evaluating patients’ experiences and fentanyl use.

**Acknowlegements**

ATP Foundation.

**References**

1. Baxter L.E., Campbell A., DeShields M., Levounis P., Martin J.A., McNicholas L., Payte J.T., Salsitz E.A., Taylor T., Wilford B.B.. Safe methadone induction and stabilization report of an expert panel.. Journal of Addiction Medicine 2013; 2013; 7:pp. 377–386.
2. Comer S.D., Cahill C.M. Fentanyl: Receptor pharmacology, abuse potential, and implications for treatment.. Neuroscience & Biobehavioral Reviews November 1 2019; 106:49–57.
3. Bisaga A., Mannelli P., Sullivan M.A., Vosburg S.K., Compton P., Woody G.E., Kosten T.R.. Antagonists in the medical management of opioid use disorders: Historical and existing treatment strategies.. The American Journal on Addictions 2018; 27:177–187.

## P19 “Yeah, this is not going to work for me.” A qualitative exploration among incarcerated individuals on methadone treatment barriers

### Justin Berk^1^, Megan Martin, MA^1^, Michael-Evans James^1^, Cameron Miller^1^, Eliana Kaplowitz^2^, Lauren Brinkley-Rubinstein, PhD^3^

#### ^1^Alpert Medical School at Brown University, Providence, Rhode Island, United States; ^2^The Fortune Society, New York, New York, United States; ^3^Population Health Sciences, Duke University School of Medicine, Durham, North Carolina, United States.

##### **Correspondence:** Justin Berk (justin_berk@brown.edu)

*Addiction Science & Clinical Practice* 2025, **20(Suppl 1)**:P19.

Berk J, Miller C, James ME, et al. “Yeah, this is not going to work for me”–The impact of federal policy restrictions on methadone continuation upon release from jail or prison. *Journal of Substance Use and Addiction Treatment*. 2025;168:209,538. dooi:0.1016/j.josat.2024.209538

## P20 A literature review of pharmacy based methadone dispensing in non-U.S. settings and the effect on patient outcomes

### Elisabel Ramirez^1^, France McGaffey MPP^2^, Noa Krawczyk PhD^3^

#### ^1^Mercy College and NYU Langone Center for Opioid Epidemiology and Policy, Bronx, New York, United States; ^2^Substance Use Prevention and Treatment Initiative, Pew Charitable Trusts, Washington D.C., United States; ^3^Department of Population Health, NYU Langone Health, New York, New York, United States

##### **Correspondence: **Elisabel Ramirez (eramirez26@mercy.edu)

*Addiction Science & Clinical Practice* 2025, **20(Suppl 1)**:P20

Introduction: In the United States, individuals are unable to receive methadone treatment for opioid use disorder (OUD) because limited options are available for methadone dosing, which happen at Opioid Treatment Programs. In other countries, pharmacies dispense methadone to those with OUD with a prescription from a qualified clinician. Examining treatment outcomes may inform U.S. strategies to increase access. We conducted a literature review of non-U.S. studies examining the effect of dispensing methadone at pharmacies on patient outcomes. Methods: PubMed and Google scholar were searched for eligible studies. We used search terms including: methadone, pharmacy, dispensing, opioid, patient, treatment, outcomes, and prescribing. Our eligibility criteria included original research studies about pharmacy dispensed methadone outside the U.S., reporting patient outcomes, and published after 1990. Results: Eleven studies met our criteria, including five cohort studies, two clinical trials, two time series analyses, one population-based study, and one cross-sectional study. Of the studies, five focused on retention in patients,^1^ who were dispensed pharmacy-based methadones,^2^ and found improved retention rates,^3^ correlated with reductions in drug usages^4^ and hospitalization, and improved mental and physical health than those dispensed in specialty care.^5^ Five focused on association between pharmacy dispensed methadone and rate of substance use incidents^6^ or deaths,^7^ finding no significant increases.^8 9 10^ One study examined differences in amount of methadone supplied in pharmacies,^11^ finding more methadone supplied during the pandemic. Discussion: Findings show pharmacy-dispensed methadone being broadly and successfully and integrated in other countries and shows promising results in patient outcomes. Pharmacy dispensing in the U.S. may increase access to treatment for OUD and improve patient outcomes. More research is needed to understand the ideal balance between observed and take-away doses of methadone in pharmacy settings.

**References**

1. Nosyk B, Sun H, Evans E, Marsh DC, Anglin MD, Hser YI, Anis AH. Defining dosing pattern characteristics of successful tapers following methadone maintenance treatment: results from a population-based retrospective cohort study. Addiction. 2012 Sep;107(9):1621–9. 10.1111/j.1360-0443.2012.03870.x. Epub 2012 May 8. PMID: 22385013; PMCID: PMC3376663.
2. Mullen, L., Barry, J., Long, J., Keenan, E., Mulholland, D., Grogan, L., & Delargy, I. (2012). A National Study of the Retention of Irish Opiate Users in Methadone Substitution Treatment. *The American Journal of Drug and Alcohol Abuse*, *38*(6), 551–558. 10.3109/00952990.2012.694516
3. Patrizia Carrieri, Laurent Michel, Caroline Lions, Julien Cohen, Muriel Vray, et al.. Methadone Induction in Primary Care for Opioid Dependence: A Pragmatic Randomized
4. Gossop M, Marsden J, Stewart D, Kidd T. The National Treatment Outcome Research Study (NTORS): 4–5 year follow-up results. *Addiction*. 2003;98(3):291–303. 10.1046/j.1360-0443.2003.00296.xTrial (ANRS Methaville). PLoS ONE, 2014, 9 (11), pp.112328–112328. ff10.1371/journal.pone.0112328ff. ffhal-01772534f
5. Gauthier G, Eibl JK, Marsh DC. Improved treatment-retention for patients receiving methadone dosing within the clinic providing physician and other health services (onsite) versus dosing at community (offsite) pharmacies. Drug and Alcohol Dependence. 2018;191:1–5. 10.1016/j.drugalcdep.2018.04.029
6. Nolan S, Hayashi K, Milloy MJ, et al. The impact of low-threshold methadone maintenance treatment on mortality in a Canadian setting. Drug and Alcohol Dependence. 2015;156:57–61. 10.1016/j.drugalcdep.2015.08.037
7. Keen J, Oliver P, Mathers N. Methadone maintenance treatment can be provided in a primary care setting without increasing methadone-related mortality: the Sheffield experience 1997–2000. *Br J Gen Pract*. 2002;52(478):387–389.
8. Gomes T, Campbell TJ, Kitchen SA, et al. Association Between Increased Dispensing of Opioid Agonist Therapy Take-Home Doses and Opioid Overdose and Treatment Interruption and Discontinuation. *JAMA*. 2022;327(9):846–855. 10.1001/jama.2022.1271
9. Lintzeris N, Deacon RM, Hayes V, et al. Opioid agonist treatment and patient outcomes during the COVID-19 pandemic in south east Sydney, Australia. Drug and Alcohol Review. 2022;41(5):1009–1019. 10.1111/dar.13382
10. Strang J, Hall W, Hickman M, Bird SM. Impact of supervision of methadone consumption on deaths related to methadone overdose (1993–2008): analyses using OD4 index in England and Scotland. BMJ. 2010;341:c4851. 10.1136/bmj.c4851
11. Kitchen SA, Campbell TJ, Men S, et al. Impact of the COVID-19 pandemic on the provision of take-home doses of opioid agonist therapy in Ontario, Canada: A population-based time-series analysis. International Journal of Drug Policy. 2022;103:103644. 10.1016/j.drugpo.2022.103644

## P21 Improving access to methadone in jails and prisons: an innovative approach

### Sara Whaley^1^, Josh Sharfstein^1^, Brendan Saloner^2^

#### ^1^Health Policy & Management, Johns Hopkins Bloomberg School of Public Health, Baltimore, Maryland, United States; ^2^Mental Health, Johns Hopkins Bloomberg School of Public Health, Baltimore, Maryland, United States

##### **Correspondence:** Sara Whaley (swhaley6@jh.edu)

*Addiction Science & Clinical Practice* 2025, **20(Suppl 1)**:P21

Providing methadone to incarcerated individuals is an evidence-based way to save lives. Despite guidance from the Department of Justice that this can be a violation of the Americans with Disabilities Act, the vast majority of correctional facilities do not provide access to this life-saving medication. Federal regulations around the dispensing of methadone are one of the major barriers to using methadone in jails and prisons. Traditionally, correctional facilities that want to provide methadone have had to either become an opioid treatment program (OTP) or contract with a community-based OTP. These two options are costly, logistically burdensome, and not well-suited for correctional settings. However, DEA regulations and guidance give another option for correctional settings-they are permitted to use methadone in the same way as hospitals. So long as the patient is receiving treatment for another medical or behavioral health condition, these facilities can use methadone in the same way that they use other controlled substances. Facilities must also be registered with the DEA and in compliance with any state laws or regulations around the use of methadone and controlled substances. In consultation with our team, facilities have written to DEA and SAMHSA to inform the agencies of their plans to provide methadone under this provision, though as of July 2023 no facilities have yet begun doing this. As a best practice, facilities that want to pursue this approach should: inform federal agencies of their intention; build a relationship with an addiction medicine provider for advice as needed; and develop protocols around the use of methadone including dosing and discharge planning. Facilities can also petition DEA for an exception that would let them use methadone to treat anyone with methadone, not just people with another diagnosis. Finally, long-term care facilities could also use this approach to provide methadone to people residing there.

**Acknowledgements**

This work was funded by Bloomberg Philanthropies as part of the Bloomberg Overdose Prevention Initiative.

## P22 Considerations in transitions from liquid to tablet methadone in persons with opioid use disorder

### Kelly Dunn^1^, Greer McKendrick^1^, Will Davis^1^, Michael Sklar^1^, Kori Kindbom^1^, Patrick Finan^2^, Denis Antoine^1^

#### ^1^Psychiatry and Behavioral Sciences, Johns Hopkins University School of Medicine, Baltimore, Maryland, United States; ^2^Department of Anesthesiology, University of Virginia, Charlottesville, Virginia, United States

##### **Correspondence:** Kelly Dunn (kdunn9@jhmi.edu)

*Addiction Science & Clinical Practice* 2025, **20(Suppl 1)**:P22

Among those with Opioid Use Disorder (OUD) that receive medication for treatment (MOUD), methadone remains the primary pharmacological treatment utilized for long-term recovery. With complications arising from stabilization of fentanyl-exposed individuals onto Suboxone (buprenorphine/naloxone) within recent years, there has been enhanced use of methadone. Methadone clinics primarily offer methadone in its liquid form, though it is also produced in tablet formulations. Although there has been considerable research on the many aspects enveloped within methadone-maintained persons (MPP), minimal efforts have directly examined the characteristics of liquid vs. tablet methadone. In this study, our primary outcome is to examine whether split methadone dosing effectively manages both OUD-related metrics and clinically significant pain compared to a single dose. Methadone is administered via electronic MedMinder pillboxes that are cellularly enabled to allow for twice-daily dosing. Eligible participants first undergo a two-week transition period in which liquid administration of methadone is discontinued while the participants begin taking methadone in its tablet formulation. To specifically monitor changes from switching the methadone formulation, at the end of the transition period participants answer an array of questions designed to characterize the experience of changing from liquid to tablet methadone administration (i.e. “How similar does the tablet form hold you in comparison to the liquid form?”; “If you have experienced any advantages from the tablets, what have they been?”; If you have experienced any disadvantages from the tablets, what have they been?”), including changes in specific OUD-related symptoms and participant preference. With this project, we aim to inform other clinicians, researchers, and MMP about common expectations encountered when transitioning from liquid to tablet methadone.

**Acknowledgements**

This study is funded by NIDA project R01DA056045 (Dunn).

